# Evaluating and Comparing the Natural Cell Structure and Dimensions of Honey Bee Comb Cells of Chinese Bee, *Apis cerana cerana* (Hymenoptera: Apidae) and Italian Bee, *Apis mellifera ligustica* (Hymenoptera: Apidae)

**DOI:** 10.1093/jisesa/ieab042

**Published:** 2021-07-02

**Authors:** Shunhua Yang, Shangkao Deng, Haiou Kuang, Danyin Zhou, Xueyang Gong, Kun Dong

**Affiliations:** Yunnan Provincial Engineering and Research Center for Sustainable Utilization of Honeybee Resources, Eastern Bee Research Institute, College of Animal Science and Technology, Yunnan Agricultural University, Kunming 650201, China

**Keywords:** honey bee, comb, structure, angle, diameter

## Abstract

The hexagonal structure of the honey bee comb cell has been the source of many studies attempting to understand its structure and function. In the storage area of the comb, only honey is stored and no brood is reared. We predicted that honey bees may construct different hexagonal cells for brood rearing and honey storage. We used quantitative analyses to evaluate the structure and function of the natural comb cell in the Chinese bee, *Apis cerana cerana* and the Italian bee, *A. mellifera ligustica*. We made cell molds using a crystal glue solution and measured the structure and inclination of cells. We found that the comb cells of *A. c. cerana* had both upward-sloping and downward-sloping cells; while the *A. m. ligustica* cells all tilted upwards. Interestingly, the cells did not conform to the regular hexagonal prism structure and showed irregular diameter sizes. In both species, comb cells also were differentiated into worker, drone and honey cells, differing in their diameter and depth. This study revealed unique differences in the structure and function of comb cells and showed that honey bees design their cells with precise engineering to increase storage capacity, and to create adequate growing room for their brood.

Honey bee combs are the most perfect structures found in nature. Over several centuries, scientists have devoted a lot of attention to analyzing the geometric structures of honey bee comb cells. Pappus, a mathematician of the Alexandrian School in ancient Greece, realized that when equilateral triangles, squares, and regular hexagons have the same perimeter, the area enclosed by the latter is largest. Honeybees take regular hexagons as the main shape of their cells and make this choice based on the storage capacity it can provide. In much later studies, Kepler (1619), a German astronomer, discovered that three rhombuses form the bottom of each cell; while Maraldi (1712), an Italian astronomer, measured the angle of these at the base of each cell, and showed that they enclosed an obtuse angle of 110° and an acute angle of 70°. Later, Réaumur, a French scientist, and Koenig, a German mathematician, calculated that when the obtuse angle of each rhombus at the base of each cell was 109° 26ʹ and when the acute angle was 70° 34ʹ, a cell would have the largest volume, smallest surface area, and therefore increased storage capacity (Réaumur et al. 1740). In subsequent work, MacLaurin (1743), a Scottish mathematician, calculated that the ideal obtuse angle of a rhombus is 109° 28ʹ, while the ideal acute angle is 70° 32ʹ, a result confirmed by later researchers (Sharpe 1828, Glaisher 1873, Hennessy 1885, Everett 1903, Graesser 1946). Mathematicians regarded these cells as theoretical constructs and, therefore, resolved the angle of the lozenges at the bottom of the cell and elaborated the theoretical structure of the cell from the perspective of saving construction materials. It is nevertheless the case that when honey bees build combs, they consider numerous aspects, including the strength, arrangement, and inclination of their cells. These considerations mean that the actual structure of cells does not meet the expectations of mathematicians. It has also been shown that the metrological abilities of honeybees are limited and so they are unable to construct completely regular hexagonal cells (Hepburn 1986, Hepburn and Whiffler 1991, Hepburn et al. 2014).

Chinese bee, *Apis cerana cerana* and Italian bee, *Apis mellifera ligustica*, are two honey bee species of the genus *Apis* originally distributed in Asia and Europe, respectively. Adult worker bees utilize the wax secreted by glands in their abdomens to build two types of hexagonal cells, one is a worker cell while the other is a drone cell. The worker cell is used to rear worker broods, store pollen, and honey, while a small number of drone cells are built to rear drone broods (Winston 1987). Indeed, thousands of cells form a comb, and several combs form a nest. However, we have observed that honey bee natural comb cells in the storage area of combs are only used for storing honey, and are never used for rearing brood. We think that these cells are neither worker cells nor drone cells, but honey cells. Therefore, we speculate that honey bees build three types of hexagonal natural comb cell: worker, drone, and honey cell. It has long been thought that the same type of cells have the same structures, and researchers are, therefore, able to exploit measuring tools to measure cell parameters (Maraldi 1712). The main parameters measured in these studies are diameter, hexagon side length, and depth of each cell. For example, the distance between the parallel walls is considered the diameter of the cell (Hepburn 1983, Hepburn et al. 2014, Svečnjak et al. 2019). Earlier work has shown that the diameter of an *Apis cerana Fabricius (Hymenoptera: Apidae)* worker cell ranges between 4.20 mm and 4.87 mm (Ruttner 1988), in contrast, the diameter of a drone cell ranges between 4.70 mm and 5.30 mm (Hepburn and Radloff 2011). The diameter of an *Apis mellifera Linnaeus (Hymenoptera: Apidae)* worker cell ranges between 4.62 mm and 5.51 mm, and the diameter of a drone cell ranges between 6.15 mm and 6.91 mm (Ruttner 1988). Recently, there has been growing interest and debate on understanding the structure, size, organization and form of the honey bee comb cells (Hepburn et al. 2014, Saucy 2014, Nazzi 2016, Svečnjak et al. 2019). Many research studies have been conducted to understand the structure, size, and mechanisms of formation, revealing a lot of data on the rules of honey bee comb cell construction(Pirk et al. 2004, Bauer and Bienefeld 2013, Karihaloo et al. 2013, Hepburn et al. 2014, Oldroyd and Pratt 2015, Nazzi 2016, Oeder and Schwabe 2017, Jeong et al. 2018, Narumi et al. 2018, Jeong et al. 2019, Talukdar and Dutta 2019).

In previous studies, researchers have used resin to make comb cell molds and observed their structure, mainly measuring structural changes with age(Pirk et al. 2004, Hepburn et al. 2007), however, these molds have not been used to measure the sizes of cell and, therefore, learn more information about the structure, organization, and function of the cells. In this research study, we performed a quantitative analysis on the structure of honey bee newly constructed natural comb cells, clarified the actual structure, organization, and function of the honey bee natural comb cells, and revealed the structural characteristics of the natural comb cells constructed by honey bees. By injecting a crystal glue solution made of epoxy resin and its curing agent into the natural newly built comb cells by *A. c. cerana* and *A. m. ligustica*, molds of natural newly built comb cells were made. We then analyzed the actual structure and size of the comb cells by (1) measuring cell inclination angles; (2) measuring and analyzing cell diameters; (3) measuring and analyzing cell depths; and (4) measuring the lengths of nine sides of three quadrilaterals at the bottom of cell molds. We predict that the results will yield insight in the comb building strategy of honey bees and the related governing rules. This not only enriches the knowledge of honey bee biology, but also provides data support for the improvement of the wax comb foundation.

## Materials and Methods

### Natural Combs Newly Built

Chinese bee (*A. c. cerana*) and Italian bee (*A. m. ligustica*) were used as experimental honey bee species, and the experimental colonies were set up with standard movable frame in Langstroth hives. Each experimental colony consisted of the same-age queen and frames fully covered with workers. For the Chinese bee, there were 15 experimental colonies, and for the Italian bee there were 20 experimental colonies. The hive of each experimental colony was divided into two areas using a vertical queen excluder. For the Chinese bee colony, each area in the hive had two wax combs where the queen laid eggs in one of two areas; an empty movable frame was added into the other area for building new natural combs. Similarly, for the Italian bee colony, there were three combs in one area for rearing broods, and there were four combs in another area where an empty frame was added for building new natural combs.

Each colony was fed with sugar syrup every evening during comb-building until the inner area of the empty frame was completely filled with the wax comb. The natural new comb was then transferred to the outside of the following board in the same hive to facilitate cleaning of the stored food by the worker bees. When the comb was clean and empty, it was taken out of the hive and sent to the laboratory to make comb cell molds.

### Cell Molds Made of Epoxy Resin

**Fig. 1.**
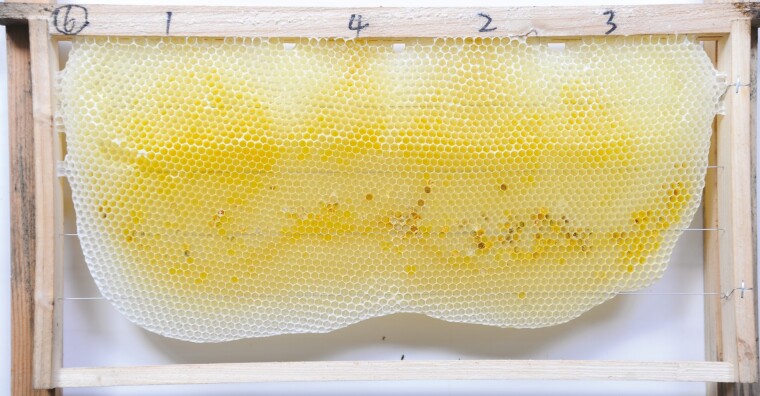
Photograph of the Chinese bee, *A. c. cerana*, honey bee natural comb.

In each honey storage area on both sides of the newly built combs, 60 hexagonal honey cells were randomly selected as the samples for making the honey cell molds. In the non-honey storage area of the comb, 60 hexagonal worker cells were randomly selected as the samples for making the worker cell molds, and 60 hexagonal drone cells were selected as the samples for making the drone cell molds. The selected cells with one vertex pointing directly upward, such that rows of cells were arranged in a vertical orientation form in a honey bee comb as shown in Fig. 1. After the frame with a new comb was placed horizontally, the crystal glue solution was prepared by mixing epoxy resin and its curing agent according to a volume ratio of 1:1. The crystal glue solution was extracted using a disposable syringe and then injected into the selected sample cells. The amount of crystal glue solution injected into the cell should be appropriate so that the fluid level was kept flush with the cell mouth. The crystal glue solution was left to solidify in the cell at room temperature for 12 h., after which it forms a fixed-shape molding to the shape and size of the comb cell. During the curing process, there was no effect of the crystal glue solution on the shape or volume of the cell; the cell retained its shape and structure, and the solution did not emit any heat that could shrink or expand the cell.

### Measurement Index of Comb Cell Mold

To measure the inclination angle of comb cells, we took the horizontal plane perpendicular to the comb as the reference system, where the acute angle formed by the six parallel sides of the hexagonal prism cell with the horizontal plane is defined as the inclination angle of the cell. Consequently, if the cell is inclined upward, the acute angle is positive, while if the cell inclined downward, the acute angle is negative. When the horizontal plane is parallel to the six parallel sides of cell hexagonal prisms, an angle of 0° is formed and the cell remains horizontal.

**Fig. 2.**
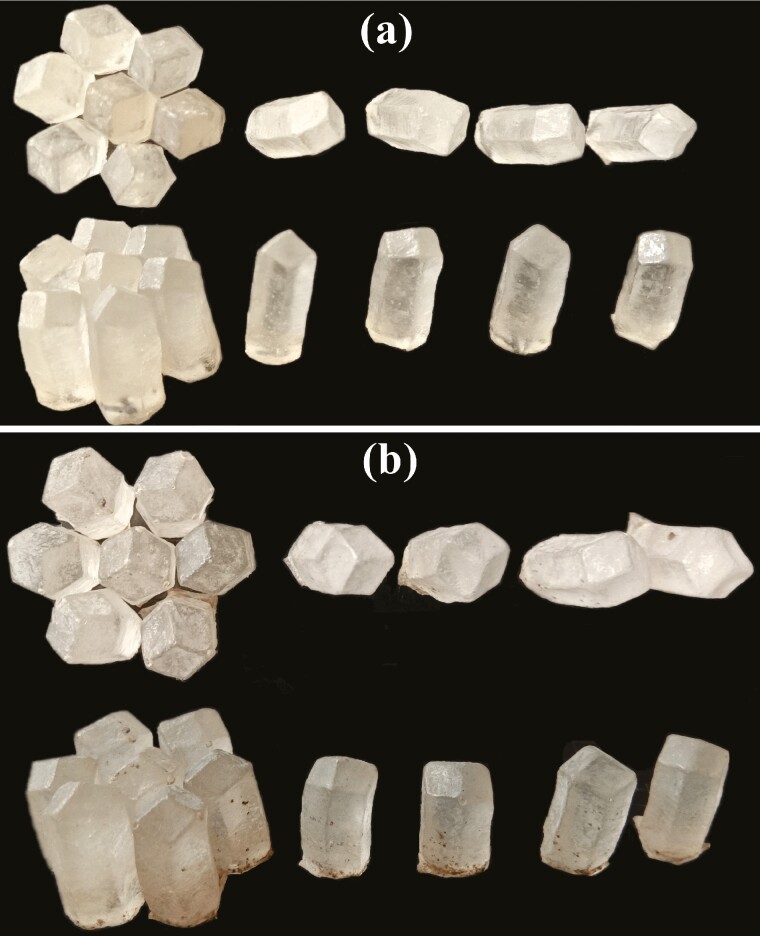
Cell molds of Chinese bee, *A. c. cerana*. (a) Molds of worker cells and, (b) Molds of drone cells.

**Fig. 3.**
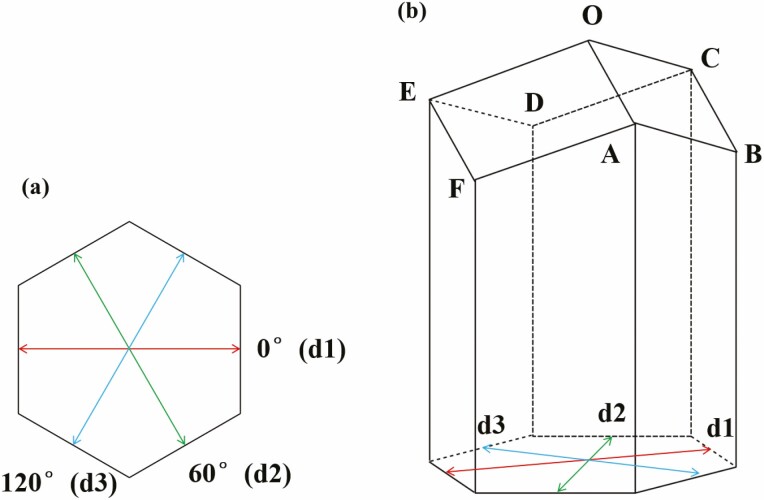
(a) The diameter of a honey bee comb cell in three directions. The diameter of a cell in 0 ° direction (***d1***: red line with an arrow), the diameter of a cell in 60 ° direction (***d2***: green line with an arrow), the diameter of a cell in 120 ° direction (***d3***: blue line with an arrow); and (b) The sides AB, BC, CD, DE, EF, FA, OC, OE and OA of three quadrilaterals (ABCO, CDEO, EFAO) of the cell base.

The inclination angle of the cells was measured from the lateral of the comb with an electronic protractor (accuracy ± 0.01°). Then, the comb was soaked in 90°C hot water to melt the beeswax, which separated the molds (Fig. 2). The hardness of the cell mold was above 80 HD, and it did not deform in 90°C hot water. Therefore, the structure and size of the cell mold can reflect the actual structure and size of the honey bee comb cell. Finally, Vernier calipers (accuracy ± 0.01 mm) were used to measure the three diameters of the cell mold: the diameter***d1*** in the 0° direction, the diameter ***d2*** in the 60° direction, and the diameter ***d3*** in the 120° direction (Fig. 3a). The height of the cell mold (i.e., cell depth) was measured using a Vernier caliper, for each of the molds: the worker cell mold ***wh***, the drone cell mold ***dh***, and the honey cell mold ***hh***. The three quadrilaterals at the bottom of the cell mold were ABCO, CDEO, and EFAO, the lengths of AB, BC, CD, DE, EF, FA, OC, OE, and OA (Fig. 3b) were measured using a Vernier caliper.

### Statistical Analysis

#### Cell Inclination Angle

Descriptive statistics were used to analyze the inclination angle of the cells, and we constructed histograms to display the frequency distribution of cell inclination angle.

#### Comparison of Diameters in Different Directions of the Same Type of Cell for the Same Honey Bee Species

One-way analysis of variance (ANOVA) was used to compare the differences in the size of the diameter of different directions in the same type of cell for the same honey bee species by comparing the diameter ***d1*** in the 0° direction, ***d2*** in the 60° direction and***d3*** in the 120° direction.

#### Comparison of Diameters of Different Types of Cell in the Same Direction for the Same Honey Bee Species

The difference in the cell diameter in the same direction of different cell types for the same honey bee species was also compared using a one-way ANOVA. The diameter ***wd1*** in the 0° direction of worker cell, the diameter***dd1*** in the 0° direction of drone cell and the diameter***hd1*** in the 0° direction of the honey cell were compared. This was done for the 60° direction and 120° direction respectively for the same species.

#### Comparison of Cell Depth Among Different Cell Types for the Same Honey Bee Species

One-way ANOVA was used to analyze the differences in cell depth across the three different cell types for the same honey bee species. The depth ***wh*** of worker cell, the depth ***dh*** of drone cell and the depth ***hh*** of honey cell were compared for the same honey bee species.

#### The Side Length of the Three Quadrilaterals of the Cell Base

The average length of sides AB, BC, CD, DE, EF, FA, OC, OE, and OA of three quadrilaterals (ABCO, CDEO, EFAO) was calculated.

All data obtained in the experiments were statistically analyzed with the Statistical Analysis System (SAS v8.0) software. It was provided by SAS Institute Inc., North Carolina, US.

## Results

### The Inclination of Comb Cells

**Fig. 4.**
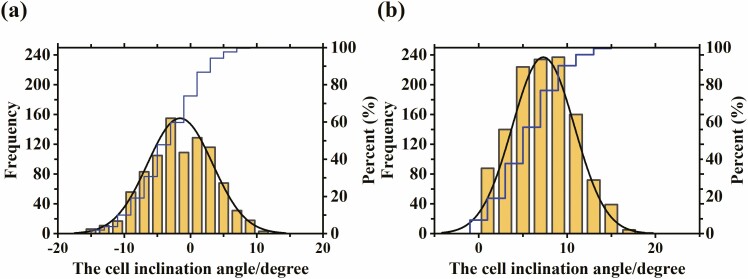
Cell inclination angle on honey bee combs. (a)The frequency distribution histogram of the cell inclination angle on Chinese bee(*A. c. cerana*) comb; (b) The frequency distribution histogram of the cell inclination angle on Italian bee(*A. m. ligustica*) comb.

We found that the honey bee comb cells of Chinese bee (*A. c. cerana*) inclined both upward and downward, while those of Italian bee (*A. m. ligustica*) only inclined upward. The upward inclination angle of Chinese bee comb cells ranged from 0.05° to 11.9°, with an average of 3.24°; while the downward inclination angle of the cells ranged from −15.1° to −0.05°, with an average of −4.8°. In *A. m. ligustica*, the upward inclination angle ranged from 0.05° to 19.25°, with an average of 7.3°. The frequency distribution histograms of the inclination angle of *A. c. cerana* and *A. m. ligustica* comb cells are shown in Fig. 4.

### Comparison of Diameters in Different Directions of the Same Type of Cell for the Same Honey Bee Species

#### A. c. cerana

**Fig. 5.**
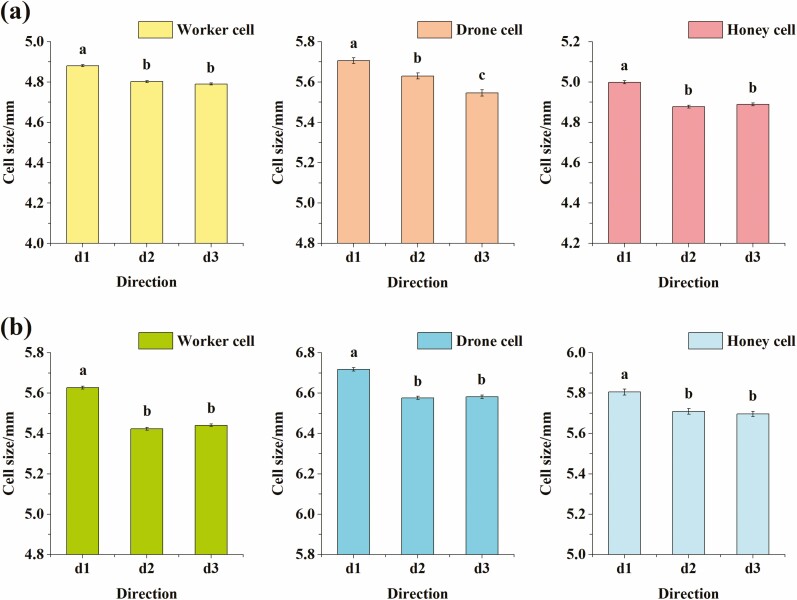
The comparison of the sizes of the diameters of honey bee comb cell in three directions. (a) The comparison of the diameters in the three directions for Chinese bee (*A. c. cerana*) worker cell or honey cell or drone cell; (b) The comparison of the diameters in the three directions for the Italian bee (*A. m. ligustica*) worker cell or honey cell or drone cell. Notes: All values in the figures are mean±standard error. The same lowercase letters among the columns of the bar graph indicate that the difference is not significant (*P* > 0.05), and different lowercase letters indicate that the difference is significant (*P* < 0.05), the same below.

The average diameter of a worker cell of the Chinese bee was 4.825 mm ± 0.003 mm; and each direction averaged as follows: 0° direction ***d1*** was 4.881 mm ± 0.005 mm, 60° direction ***d2*** was 4.802 mm ± 0.005 mm, and 120° direction ***d3*** was 4.790 mm ± 0.005 mm. The direction significantly influenced the diameter of the cell (one-way ANOVA, *F* = 100.58; df = 2, 2343; *P* < 0.0001). The results of multiple comparisons showed that the diameter ***d1*** was significantly larger than ***d2*** and ***d3*** (*P* < 0.0001), and the difference between***d2*** and ***d3*** (*P* = 0.0895 > 0.05,) was not significant (Fig. 5a). Among them, the diameter ***d1*** was the largest, followed by ***d2***, and ***d3*** was the smallest.

Similarly, the diameter size of the drone cell was significantly influenced by the direction (one-way ANOVA, *F* = 28.69; df = 2, 495; *P* < 0.0001). The average diameter of drone cell of Chinese bee was 5.627 mm ± 0.009 mm; and the average diameter at each direction: 0° direction ***d1*** was 5.706 mm ± 0.014 mm, 60° direction ***d2*** was 5.630 mm ± 0.015 mm, and 120° direction ***d3*** was 5.546 mm ± 0.016 mm. The results of multiple comparisons showed that the differences among ***d1***, ***d2,*** and ***d3*** were significant (*P* < 0.0001, Fig. 5a), with the diameter ***d1*** being the largest, followed by ***d2***, and ***d3*** was the smallest.

The honey cell diameter size was also influenced by the direction (one-way ANOVA, *F* = 91.72; df = 2, 2697; *P* < 0.0001). The average diameter of the honey cell of Chinese bee was 4.922 mm ± 0.004 mm, and at each direction, the diameter was: 0° direction***d1*** was 4.999 mm ± 0.008 mm, 60° direction ***d2*** was 4.878 mm ± 0.007 mm, and 120° direction ***d3*** was 4.889 mm ± 0.007 mm. The results of multiple comparisons showed that the diameter ***d1*** was significantly larger than ***d2*** and ***d3*** (*P* < 0.0001), and the difference between ***d2*** and ***d3*** (*P* = 0.2526 > 0.05) was not significant (Fig. 5a); the diameter ***d1*** was the largest, followed by ***d3***, and ***d2*** was the smallest.

#### A. m. ligustica

The average diameter of worker cell of Italian bee was 5.497 mm ± 0.004 mm, and was significantly influenced by the direction (one-way ANOVA, *F* = 284.89; df = 2, 2901; *P* < 0.0001). At each direction the diameter size was as follows: 0° direction ***d1*** was 5.627 mm ± 0.007 mm, 60° direction ***d2*** was 5.423 mm ± 0.007 mm, and 120° direction ***d3*** was 5.441 mm ± 0.007 mm. The results of multiple comparisons showed that the diameter ***d1*** was significantly larger than ***d2*** and ***d3*** (*P* < 0.0001), and the difference between ***d2*** and ***d3*** (*P* = 0.0594 > 0.05) was not significant (Fig. 5b); diameter***d1*** was the largest, followed by ***d3***, and ***d2*** was the smallest.

The average size diameter of drone cell of Italian bee was 6.626 mm ± 0.005 mm, and was significantly influenced by the direction of the diameter (one-way ANOVA, *F* = 95.45; df = 2, 3243; *P* < 0.0001). At each direction, the average diameter size was: 0° direction ***d1*** was 6.718 mm ± 0.008 mm, 60° direction ***d2*** was 6.577 mm ± 0.008 mm, and 120° direction ***d3*** was 6.582 mm ± 0.008 mm. The results of multiple comparisons showed that the diameter ***d1*** was significantly larger than ***d2*** and ***d3*** (*P* < 0.0001), and the difference between ***d2*** and ***d3*** (*P* = 0.7028 > 0.05) was not significant (Fig. 5b). The diameter ***d1*** was the largest, followed by ***d3***, and ***d2*** was the smallest.

Similarly, the diameter size of honey cells averaged 5.738 mm ± 0.008 mm, and was significantly influenced by the direction of the cell diameter (one-way ANOVA, *F* = 17.87; df = 2, 1140; *P* < 0.0001). The average diameter at each direction was: 0° direction ***d1*** was 5.806 mm ± 0.015 mm, 60° direction ***d2*** was 5.710 mm ± 0.014 mm, 120° direction ***d3*** was 5.697 mm ± 0.013 mm. The results of multiple comparisons showed that the diameter ***d1*** was significantly larger than ***d2*** and ***d3*** (*P* < 0.0001), and the difference between ***d2*** and ***d3*** (*P* = 0.5117 > 0.05) was not significant (Fig. 5b); the diameter ***d1*** was the largest, followed by ***d2***, and ***d3*** was the smallest.

### Comparison of Diameters of Different Types of Cell in the Same Direction for the Same Honey Bee Species

#### A. c. cerana

**Fig. 6.**
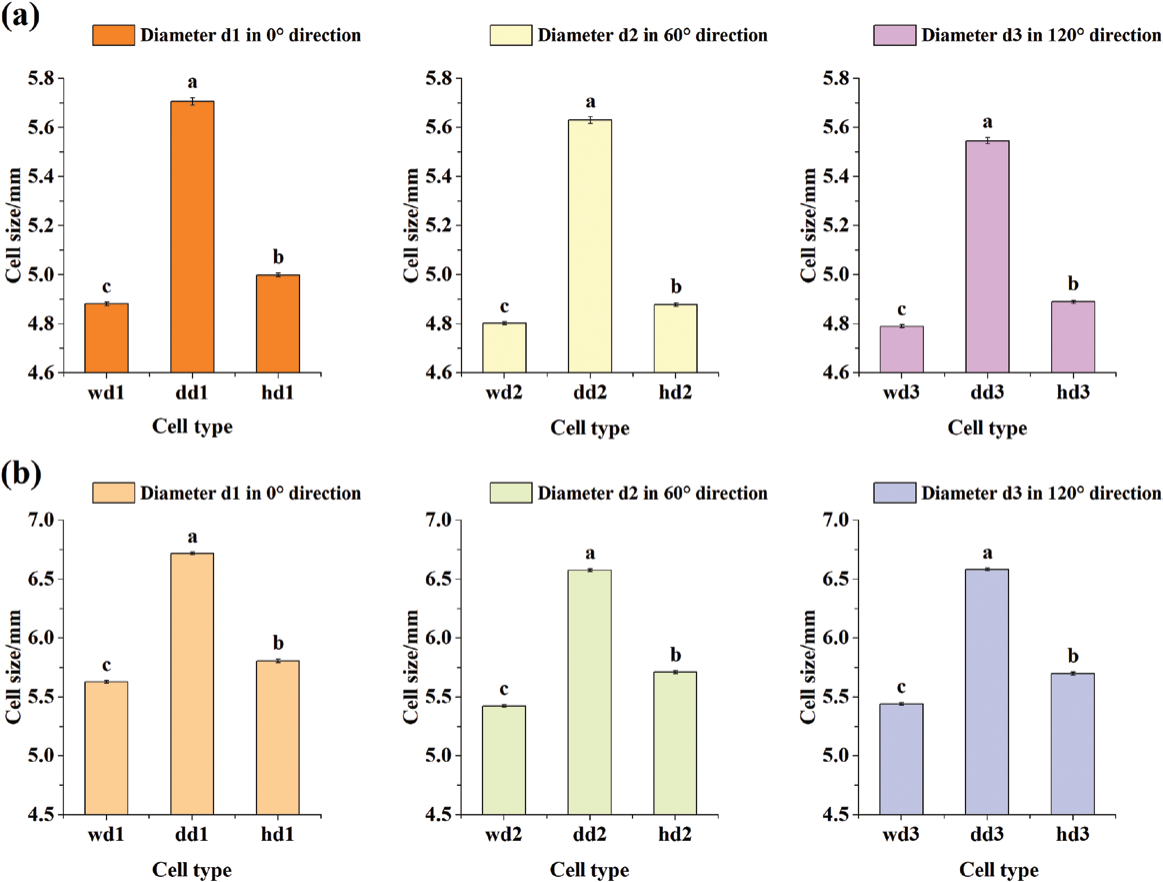
(a) The diameter *wd1* in 0° direction of worker cell of Chinese bee (*A. c. cerana*), the diameter *dd1* in 0° direction of drone cell and the diameter *hd1* in 0° direction of the honey cell were compared. Similarly, compare the diameters *wd2*, *dd2*, *hd2* in the 60° direction of the three cell types, and compare the diameters *wd3*, *dd3*, *hd3* in the 120° direction; (b) The diameter *wd1* in 0° direction of worker cell of Italian bee (*A. m. ligustica*), the diameter *dd1* in 0° direction of drone cell and the diameter *hd1* in 0° direction of the honey cell were compared. Similarly, compare the diameters *wd2*, *dd2*, *hd2* in the 60° direction of the three cell types, and compare the diameters *wd3*, *dd3*, *hd3* in the 120° direction.

Under the 0° direction, the diameter ***d1*** differed significantly across the three cell types (one-way ANOVA, *F* = 1, 233.25; df = 2, 1845; *P* < 0.0001). The average diameter of the worker cell of Chinese bee ***wd1*** was 4.881 mm ± 0.007 mm, that of the drone cell ***dd1*** was 5.706 mm ± 0.015 mm, and that of the honey cell ***hd1*** was 4.999 mm ± 0.007 mm. The results of multiple comparisons showed that the differences among diameters ***wd1***, ***dd1,*** and ***hd1*** were significant (*P* < 0.0001, Fig. 6a); ***dd1*** was the largest, followed by ***hd1***, ***wd1*** was the smallest.

Under the 60° direction, the diameter ***d2*** differed significantly across cell types (one-way ANOVA, *F* = 1,588.91; df = 2, 1845; *P* < 0.0001). The average diameter of the worker cell of Chinese bee ***wd2*** was 4.802 mm ± 0.006 mm, that of the drone cell ***dd2*** was 5.630 mm ± 0.014 mm, and that of the ***hd2*** was 4.878 mm ± 0.006 mm. The results of multiple comparisons showed that the differences among diameters ***wd2***, ***dd2,*** and ***hd2*** were significant (*P* < 0.0001, Fig. 6a); ***dd2*** was the largest, followed by ***hd2***, ***wd2*** was the smallest.

Under the 120° direction, the diameter ***d3*** was significantly different across cell types (one-way ANOVA, *F* = 1,313.96; df = 2, 1845; *P* < 0.0001). The average diameter of the worker cell of Chinese bee ***wd3*** was 4.790 mm ± 0.006 mm, that of the drone cell ***dd3*** was 5.546 mm ± 0.013 mm, and that of the honey cell ***hd3*** was 4.889 mm ± 0.006 mm. The results of multiple comparisons showed that the differences among diameters ***wd3***, ***dd3,*** and ***hd3*** were significant (*P* < 0.0001, Fig. 6a); ***dd3*** was the largest, followed by ***hd3***, ***wd3*** was the smallest.

#### A. m. ligustica

Under the 0° direction, the diameter ***d1*** was significantly different across cell types (one-way ANOVA, *F* = 5,154.89; df = 2, 2428; *P* < 0.0001).The average diameter of the worker cell of Italian bee ***wd1*** was 5.627 mm ± 0.008 mm, that of the drone cell ***dd1*** was 6.718 mm ± 0.008 mm, and that of the honey cell ***hd1*** was 5.806 mm ± 0.013 mm. The results of multiple comparisons showed that the differences among diameters ***wd1***, ***dd1,*** and***hd1*** were significant (*P* < 0.0001, Fig. 6b); ***dd1*** was the largest, followed by ***hd1***, ***wd1*** was the smallest.

Under the 60° direction, the diameter ***d2*** was significantly different across all cell types (one-way ANOVA, *F* = 6,164.92; df = 2, 2428; *P* < 0.0001). The average diameter of the worker cell of Italian bee ***wd2*** was 5.423 mm ± 0.008 mm, that of the drone cell ***dd2*** was 6.577 mm ± 0.007 mm, and that of the honey cell ***hd2*** was 5.710 mm ± 0.012 mm. The results of multiple comparisons showed that the differences among diameters ***wd2***, ***dd2,*** and ***hd2*** were significant (*P* < 0.0001, Fig. 6b); ***dd2*** was the largest, followed by ***hd2***, ***wd2*** was the smallest.

Under the 120° direction, the diameter ***d3*** differed significantly across the three cell types (one-way ANOVA, *F* = 5,743.53; df = 2, 2428; *P* < 0.0001). The average diameter of the worker cell of Italian bee ***wd3*** was 5.441 mm ± 0.008 mm, that of the drone cell ***dd3*** was 6.582 mm ± 0.008 mm, and that of the honey cell ***hd3*** was 5.697 mm ± 0.013 mm. The results of multiple comparisons showed that the differences among diameters ***wd3***, ***dd3,*** and ***hd3*** were significant (*P* < 0.0001, Fig. 6b); ***dd3*** was the largest, followed by ***hd3***, ***wd3*** was the smallest.

### Comparison of the Depth of Different Types of Cell

**Fig. 7.**
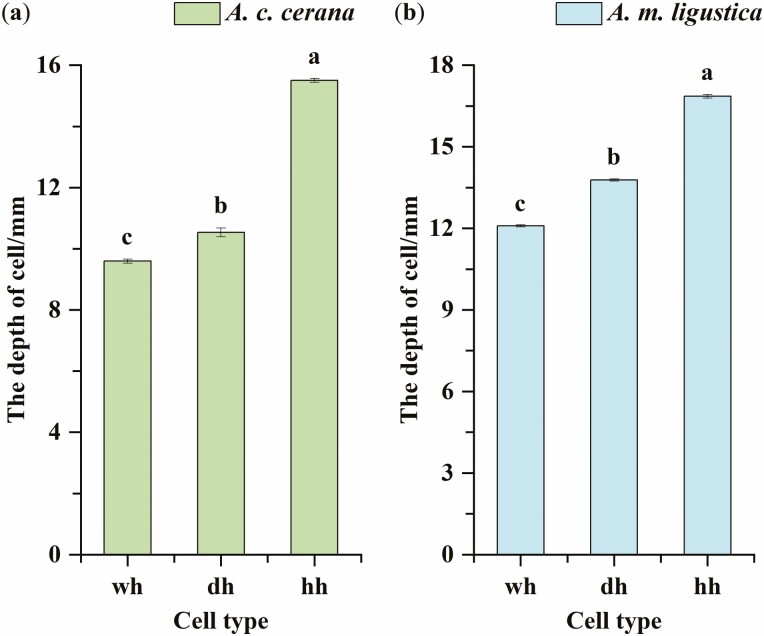
(a) A size comparison of the depth among different cell types of Chinese bee(*A. c. cerana*) or (b) Italian bee(*A. m. ligustica*) comb cells.

We found significant differences across the depth of the different types of cell for Chinese bee (one-way ANOVA, *F* = 2, 233.98; df = 2, 1845; *P* < 0.0001). The average depth of the worker cell ***wh*** was 9.595 mm ± 0.066 mm, that of the drone cell ***dh*** was 10.533 mm ± 0.144 mm, and that of the honey cell ***hh*** was 15.499 mm ± 0.062 mm. The results of multiple comparisons showed that the differences among depths ***wh***, ***dh,*** and ***hh*** were significant (*P* < 0.0001, Fig. 7a); ***hh*** was the largest, followed by ***dh***, ***wh*** was the smallest.

Similarly in the Italian bee, there were significant differences in the depth of different types of cell (one-way ANOVA, *F* = 2,001.31; df = 2, 2428; *P* < 0.0001). The average depth of the worker cell ***wh*** was 12.088 mm ± 0.040 mm, that of the drone cell ***dh*** was 13.776 mm ± 0.038 mm, and that of the honey cell ***hh*** was 16.857 mm ± 0.064 mm. The results of multiple comparisons showed that the differences among depths ***wh***, ***dh,*** and ***hh*** were significant (*P* < 0.0001, Fig. 7b); ***hh*** was the largest, followed by ***dh***, ***wh*** was the smallest.

### The Side Length of the Three Quadrilaterals of the Cell Base

For the side length of the three quadrilaterals of the cell base of Chinese bee, the statistical results showed that the average side length (AB, BC, CD, DE, EF, FA, OC, OE, OA) of the three quadrilaterals of worker cell base was 2.769 mm ± 0.006 mm, and the average side length (AB, BC, CD, DE, EF, FA, OC, OE, OA) of the three quadrilaterals of drone cell base of Chinese bee was 3.191 mm ± 0.015 mm. For the side length of the three quadrilaterals of the cell base of Italian bee, the statistical results showed that the average side length (AB, BC, CD, DE, EF, FA, OC, OE, OA) of the three quadrilaterals of worker cell base was 3.144 mm±0.006 mm, and the average side length (AB, BC, CD, DE, EF, FA, OC, OE, OA) of the three quadrilaterals of drone cell base of Italian bee was 3.696 mm±0.007 mm. The average lengths of AB, BC, CD, DE, EF, FA, OC, OE, and OA are shown in Table 1.

**Table 1.** The average side length of the cell base (mean ± S.E., Unit: mm)

		*A. c. cerana*		*A. m. ligustica*	
		Cell type		Cell type	
Quadrilateral	Side	Worker cell	Drone cell	Worker cell	Drone cell
ABCO	AB	2.743 ± 0.019	3.185 ± 0.035	3.154 ± 0.018	3.695 ± 0.020
	BC	2.787 ± 0.018	3.160 ± 0.047	3.095 ± 0.020	3.742 ± 0.023
	OC	2.789 ± 0.018	3.220 ± 0.041	3.215 ± 0.016	3.715 ± 0.019
	OA	2.761 ± 0.020	3.142 ± 0.035	3.157 ± 0.080	3.718 ± 0.019
CDEO	CD	2.787 ± 0.019	3.260 ± 0.055	3.160 ± 0.019	3.647 ± 0.018
	DE	2.775 ± 0.018	3.217 ± 0.051	3.112 ± 0.018	3.689 ± 0.020
	OC	2.789 ± 0.018	3.220 ± 0.041	3.215 ± 0.016	3.715 ± 0.019
	OE	2.760 ± 0.016	3.184 ± 0.030	3.216 ± 0.018	3.688 ± 0.018
EFAO	EF	2.736 ± 0.019	3.117 ± 0.042	3.037 ± 0.020	3.677 ± 0.021
	FA	2.783 ± 0.020	3.238 ± 0.062	3.147 ± 0.021	3.694 ± 0.019
	OA	2.761 ± 0.020	3.142 ± 0.035	3.157 ± 0.080	3.718 ± 0.019
	OE	2.760 ± 0.016	3.184 ± 0.030	3.216 ± 0.018	3.688 ± 0.018

## Discussion

The honey bee cell is an engineering wonder, and has been the source of much research over time. Researchers mostly attempt to understand the structure and function, and storage capabilities of the honey bee cell. Here we show that honey bees are able to construct honey bee comb cells different in structure and function. We found significant differences in honey bee comb cells for honey storage, and rearing brood (workers and drone). Moreover, the depth of different types of cell differs significantly. Previous studies have revealed that the depth and diameter can influence the storage capacity of the honey comb cells.

Cell inclination angles, the inclination of the cells from the plane perpendicular to the comb midrib, have been widely reported for honey bee, *A. mellifera*. Huber (1814) found comb cells tilted upward with average values ranging from 4° to 5° and a maximum cell inclination angles of greater than 20°. Some researchers reported upwards of 16°(Vogt 1911), while others found worker cells to have an average of 5° and a range of 0–13°, drone cells to have an average of 10.7° and a range of 9–12°(Taber and Owens 1970). Dadant (1975) and Lindauer and Nedel (1959) reported angles ranging from 9° to 14°. Vandenberg et al. (1985) had shown that the measurements ranged from 4° to 13°. In most studies, cells were shown to tilt upwards, but some research shows evidence for downward tilt for *A. m. ligustica* (Gontarski 1935). Cell inclination angles of *A. cerana* had been measured previously by investigators. The natural comb cells of *A. cerana* inclined 11° upward (Wahyudi 2018).

Several works of literature have recorded that the cells incline upward to prevent honey from leaking out of the cells (Frisch 1969, Gallo and Chittka 2018, Wahyudi 2018). However, the research results of Oeder and Schwabe (2020) showed that the benefit for the upward tilt of the cells for the bees was not to prevent leakage of honey, nor was it a necessary condition for breeding bees, but to direct about 10% of the weight of cell contents onto the mid wall, thus increasing the carrying capacity of the comb. In this study, we found that the natural comb cells of *A. c. cerana* could be upward tilted or downward tilted. However, even though the cells were tilted downwards, the honey, royal jelly, and larvae in the cells will not fall out of the cells because of the adhesive forces between the honey and the cell wall, the small diameter of the cell, and the surface tension of the honey (Guo et al. 2015, Oeder and Schwabe 2020). The capillary forces resulting from the combination of these factors prevent the honey from flowing out because of the negative pressure at the bottom of the cell.

We speculated that the downward tilted cells were easy to be turned into the emergent cells when the colony lost the existing queen bee. Studies have shown that oligoMRJP1 in complex with apisimin acts as a structural protein, ensuring that the viscosity of royal jelly is such that the queen larvae are retained in position in their vertically oriented cells to complete their development (Buttstedt et al. 2018, Pirk 2018). Although studies have shown that the direction of cell inclination is controlled by gravity (Martin and Lindauer 1966, Vandenberg et al. 1985, Oeder and Schwabe 2020), the specific mechanism is still unclear.

Based on the views of the above researchers, we speculate preliminarily when the cell is inclined downward, honey stored in it will not flow out of the cell and the brood in it can develop normally. Even if the bee colony lost its queen, the downward tilted cell is easy to be turned into the emergent cell.

In terms of diameter differences across the different directions (0°, 60°, 120°), the diameter direction has a significant effect on the diameter of comb cells. This shows that the comb cell is not a regular hexagonal prism, but it only looks like a regular hexagonal prism. In this regard, there is a view that bees build beeswax secreted by their wax glands into an array of closed-packed cylinders, which are in contact with each other, and under the heat generated by bees, the beeswax reaches a liquid equilibrium state. The adjacent cylindrical cells are connected in the triple junction to each other, and the circular cells rapidly transform into hexagonal structures (Pirk et al. 2004, Karihaloo et al. 2013, Oldroyd and Pratt 2015, Talukdar and Dutta 2019). According to Pirk et al. (2004) and Karihaloo et al. (2013), thermoplasticity and surface tension of beeswax play a key role in this process.

Combined with our results, we hypothesize that in the process of changing from a circle to a hexagon, the cell does not become a regular hexagon, but only a hexagon due to the typical form. Further, we show that measurement issues can arise due to the irregular shape of the prism in the comb cells. We suggest that researchers studying honey bee comb structure should measure the diameter of the cell in three directions (0 °, 60 ° and 120 °) so that the experimental data are more accurate. Only measuring the diameter in the direction of 0 ° will lead to larger value; only measuring the diameter in the direction of 60 ° or 120 ° will lead to a smaller value, resulting in incorrect estimations.

We found that honey bee comb cells are differentiated into worker, drone and honey cells; differing in their diameter and depth. Honey comb cells are larger and have a larger storage capacity than worker and drone cells. Honey cells are located in the upper regions of the combs(Seeley and Morse 1976). The depth of the honey cells determines the contact area between the upper edge of the combs and the support of the nest when honeybees build their nest in the wild, which undoubtedly enhances the strength and stability of the combs.

It is clear that honey bees use the structure of the honey cell economically and for storage capacity relative to the brood cells. Further, we found that the unique structure of honey bee comb cells allows for bees to perform various functions, and strengthens the storage capacity due to the depths of the cells, and separating the brood cells from the honey cells. We have gained significant insight on the structural biology of the honey bee comb, and the form, structure, and function.

## Conclusions

We found that the natural comb cells of *A. c. cerana* had both upward-sloping and downward-sloping cells; while the *A. m. ligustica* comb cells all tilted upwards. The comb cell is not a hexagonal prism based on diameter sizes of the cell in three directions, it just looks like one. In both species, the natural comb cells not only differentiated into the worker cell, drone cell, but also differentiated into the honey cell, which is beneficial to rearing brood and storing honey. The structure and size of the cell mold made from epoxy resin and its curing agent in this experiment accurately reflect the actual structure and size of the cell. We think using the cell mold is a good method to study the structure and size of honey bee comb cell.
